# Aberrant cerebellar connectivity in motor and association networks in schizophrenia

**DOI:** 10.3389/fnhum.2015.00134

**Published:** 2015-03-18

**Authors:** Ann K. Shinn, Justin T. Baker, Kathryn E. Lewandowski, Dost Öngür, Bruce M. Cohen

**Affiliations:** ^1^Schizophrenia and Bipolar Disorder Program, Psychotic Disorders Division, McLean HospitalBelmont, MA, USA; ^2^Department of Psychiatry, Harvard Medical SchoolBoston, MA, USA

**Keywords:** cerebellum, schizophrenia, functional connectivity, networks, motor, association cortex

## Abstract

Schizophrenia is a devastating illness characterized by disturbances in multiple domains. The cerebellum is involved in both motor and non-motor functions, and the “cognitive dysmetria” and “dysmetria of thought” models propose that abnormalities of the cerebellum may contribute to schizophrenia signs and symptoms. The cerebellum and cerebral cortex are reciprocally connected via a modular, closed-loop network architecture, but few schizophrenia neuroimaging studies have taken into account the topographical and functional heterogeneity of the cerebellum. In this study, using a previously defined 17-network cerebral cortical parcellation system as the basis for our functional connectivity seeds, we systematically investigated connectivity abnormalities within the cerebellum of 44 schizophrenia patients and 28 healthy control participants. We found selective alterations in cerebro-cerebellar functional connectivity. Specifically, schizophrenia patients showed decreased cerebro-cerebellar functional connectivity in higher level association networks (ventral attention, salience, control, and default mode networks) relative to healthy control participants. Schizophrenia patients also showed increased cerebro-cerebellar connectivity in somatomotor and default mode networks, with the latter showing no overlap with the regions found to be hypoconnected within the same default mode network. Finally, we found evidence to suggest that somatomotor and default mode networks may be inappropriately linked in schizophrenia. The relationship of these dysconnectivities to schizophrenia symptoms, such as neurological soft signs and altered sense of agency, is discussed. We conclude that the cerebellum ought to be considered for analysis in all future studies of network abnormalities in SZ, and further suggest the cerebellum as a potential target for further elucidation, and possibly treatment, of the underlying mechanisms and network abnormalities producing symptoms of schizophrenia.

## Introduction

Schizophrenia (SZ) is a devastating illness characterized by a wide-ranging constellation of symptoms, including hallucinations in any sensory modality, delusions of various contents and forms, irrational and idiosyncratic thinking, disorganized behavior, and negative symptoms (i.e., a decrease or -absence of thoughts, feelings, and actions). A host of other disturbances of the nervous system and its regulation, not designated by the Diagnostic and Statistical Manual of Mental Disorders (APA, 2013) as nuclear SZ symptoms, are also well-documented in SZ and include disturbances as varied as altered mood (Sim et al., 2004; Conley et al., 2007; Buckley et al., 2009), cognitive deficits (Gold and Harvey, 1993; Reichenberg and Harvey, 2007), anomalous self-experiences (Parnas et al., 2005), social dysfunction (Pinkham, 2014), and neurological soft signs (Heinrichs and Buchanan, 1988; Bombin et al., 2005; Whitty et al., 2009). The latter term describes non-localizing, non-diagnostic abnormalities in the neurological exam that reflect impairments in coordination, motor, sensory, and integrative functions (Whitty et al., 2009). While some symptoms are subtle relative to the hallmark aberrations of thought, perception, and behavior, it is clear that the clinical manifestations of SZ cut across multiple sign and symptom dimensions that include affective, cognitive, perceptual, behavioral, and motor domains.

Accommodating all of these anomalies, Andreasen hypothesized that the multitude and diversity of symptoms in SZ may be tied to a unitary pathophysiology involving misconnections within cortico-cerebellar-thalamic-cortical circuits (CCTCC), resulting in “cognitive dysmetria,” or incoordination of mental activity (Andreasen, 1999; Andreasen et al., 1999). This theory drew on earlier studies of abnormal structure and connectivity between prefrontal cortex, thalamus, and cerebellum in post-mortem and *in vivo* brain imaging studies of SZ (Andreasen et al., 1996, 1998). According to Andreasen's model, CCTCC-mediated asynchrony manifests as a “fragmented phrene” and constitutes the fundamental dysfunction underlying the phenotype of SZ (Andreasen, 1999; Andreasen et al., 1999). Independently, Schmahmann proposed a “dysmetria of thought” model, in which various neuropsychiatric conditions, including psychotic disorders, may reflect abnormal modulation of cognitive and affective processes by the cerebellum (Schmahmann, 1991, 1998). According to this view, the cerebellum, when it is functioning properly, “detects, prevents, and corrects mismatches” between intended and perceived outcomes for mental or cognitive processes in the same way it does for movement (Schmahmann, 1998). When cerebellar function is disrupted, there is “unpredictability to social and societal interaction, a mismatch between reality and perceived reality, and erratic attempts to correct the errors of thought or behavior” (Schmahmann, 1998).

Before these models were proposed, the cerebellum was thought to be involved exclusively in the planning and execution of motor activities. This classical view held that while the cerebellum received inputs from widespread cortical areas, it projected solely to primary motor cortex; i.e., information from frontal, parietal, temporal, and occipital cortices was believed to be integrated entirely for motor control (Strick et al., 2009). However, it is now well-recognized that the cerebellum is extensively connected to higher-level association cortices, and that it contributes to non-motor as well as motor functions (Stoodley and Schmahmann, 2009; Strick et al., 2009; Bostan et al., 2013; Buckner, 2013).

Notably, in patients with SZ, the cerebellum shows abnormal activation during various cognitive tasks (see Hoppenbrouwers et al., 2008; Picard et al., 2008; Lungu et al., 2013 for reviews). However, while many functional activation studies detect cerebellar abnormalities in SZ, and the cerebellum has an elaborate substructure, the available literature often refers, very generally, to activity of the cerebellum, as a whole. Published papers rarely mention what specific regions of the cerebellum are affected. It is known that the cerebellum is not a single homogeneous unit, but rather a complex system made up of multiple parallel networks that are highly interconnected with the cerebral cortex and also with thalamus and other subcortical brain regions. The cerebellum and cerebral cortex are densely and reciprocally connected in a series of parallel closed-circuit loops that repeat throughout the cerebellum and are modular, with circuits specific for association cortices vs. motor and somatosensory cortices (Schmahmann and Pandya, 1997; D'angelo and Casali, 2013). The most direct evidence for the modular organization of cerebro-cerebellar loops comes from viral transneuronal tracer studies showing that regions of the cerebellar cortex that receive input from the primary motor cortex are the same as those that project to primary motor cortex, while regions of the cerebellar cortex that receive input from prefrontal cortex (area 46) are the same as those that project to prefrontal cortex (Kelly and Strick, 2003). Given this modular architecture, and reflecting the role of the cerebellum in modulating and coordinating cerebral activity, it is not surprising that the cerebellum has a topography that generally mirrors that of the cerebrum (Buckner and Krienen, 2013). This tight relationship between cerebellum and cerebrum leads to the prediction that cerebral cortical abnormalities observed in patients with SZ might also be present in the cortex of the cerebellum.

While many studies of cerebellar-cerebral connectivity have been performed during cognitive or behavioral tasks, such tasks do not sample all relevant networks. Notably, resting state fMRI (rsfMRI) is a powerful tool for studying cerebro-cerebellar connectivity (Buckner, 2013). Communication between the cerebral cortex and cerebellum is indirect, with cerebral cortical signals relayed to the pons before they reach the cerebellum, and cerebellar outputs projecting to the thalamus before being communicated to the cerebral cortex. While the polysynaptic nature of cerebro-cerebellar circuitry has limited the application of traditional retrograde tracer techniques, rsfMRI enables investigation of connectivity patterns that are informed by, but not necessarily confined to, direct monosynaptic connections (Buckner, 2013). Furthermore, rsfMRI allows investigations of cerebro-cerebellar circuits in humans *in vivo* and in the absence of a task, which constrains evaluation to specific functional networks, can limit the participation of more severely ill patients, and is subject to differential performance due to illness, which can confound comparisons between patient and control groups. Importantly, rsfMRI enables the study of cerebro-cerebellar connectivity at the network level, allowing the characterization of functional relationships between multiple distributed brain regions.

To define the observable networks, Yeo and colleagues analyzed rsfMRI data from 1000 healthy subjects, using a clustering strategy to parcellate the cerebral cortex into intrinsic components on the basis of territories sharing similar functional connectivity (FC) profiles to other regions of cortex. They found that either 7 or 17 distinct networks provide relatively stable parcellation solutions (Yeo et al., 2011). This cortical parcellation strategy is particularly compelling, as compared with other functional parcellation strategies (e.g., independent component analysis), in that it does not require any manual removal of noise-related components, and replicates well in both healthy and patient samples (e.g., Baker et al., 2014). Using the same 1000 subject data set and cortical parcellation solutions as a reference, Buckner and colleagues subsequently developed a parcellation of the human cerebellum, by assigning every cerebellar voxel to its most strongly associated cortical network using a winner-take-all approach (Buckner et al., 2011). They found that the human cerebellum possesses a roughly homotopic map of the cerebral cortex and that the majority of the cerebellar cortex is connected to cerebral association networks. Corroborating and extending evidence from anatomical studies (Adrian, 1943; Snider and Stowell, 1944), the authors found that the cerebellum contains at least two homotopic maps of each network: an inverted representation in the anterior cerebellum and a mirror image representation in the posterior cerebellum (Buckner et al., 2011). Other rsfMRI studies of cerebro-cerebellar connectivity in healthy humans, which used different analysis techniques provide remarkably similar network topography in the cerebellum (Habas et al., 2009; Dobromyslin et al., 2012).

Our group recently investigated changes in cerebral cortical network architecture in psychosis patients, using resting state FC among nodes of the Yeo et al. cortical parcellations (Baker et al., 2014). This work, which added to the many previous studies of cerebral connectivity abnormalities in SZ, was the first to use a comprehensive and well-defined functional parcellation scheme (i.e., Yeo et al., 2011) to describe cortical network changes in psychosis. In particular, we found disrupted FC among nodes of the frontoparietal control network, with evidence for reduced network segregation between default and frontoparietal control networks (Baker et al., 2014). Given the modular closed-loop architecture of cerebro-cerebellar circuitry, the results suggest that the cortical connectivity anomalies described in SZ by our group (Baker et al., 2014) will be mirrored in the cerebellum. Hence, we expect to find altered cerebro-cerebellar FC in association networks, especially frontoparietal control networks, in SZ.

As a background to extending our analyses to cerebellum, it should be noted that several rsfMRI studies, using different methods, have already identified FC abnormalities involving the cerebellum in SZ (Liang et al., 2006; Bluhm et al., 2007; Shen et al., 2010; Collin et al., 2011; Liu et al., 2011; Repovs et al., 2011; Chen et al., 2013; Su et al., 2013). Five of these studies determined FC patterns associated with specific seeds within the cerebellum. Liang and colleagues examined global connectivity of 26 cerebellar and 90 cerebral brain regions parcellated according to the automated anatomic labeling (AAL) template (Tzourio-Mazoyer et al., 2002), and observed that many of the 158 instances of decreased connectivity and the majority of the 19 instances of increased connectivity in SZ involved the cerebellum (Liang et al., 2006). However, these authors did not further indicate the specific regions within the cerebellum that were abnormally connected in SZ. Collin and colleagues used the same anatomic parcellation of 26 cerebellar and 90 cerebral regions in SZ and unaffected siblings, calculating both global connectivity for each cerebellar region of interest (ROI) as well as FC of each discrete cerebello-cerebral connection (Collin et al., 2011). They found SZ patients to have reduced global connectivity of the right anterior cerebellum, reduced FC between right anterior cerebellum and left frontal regions and thalamus, and reduced FC between anterior cerebellar vermis and left hippocampus and thalamus (Collin et al., 2011). The anatomical parcellation scheme used by both the Liang et al. and Collin et al. studies provides valuable data but does not take into consideration the topography of intrinsic large-scale functional networks.

Liu and colleagues used the left and right cerebellum, each in its entirety, as seeds for FC analysis and found decreased negative left cerebellum seeded FC with left middle temporal gyrus, cingulate cortex, and right paracentral lobule, and decreased negative right cerebellum seeded FC with right thalamus and cingulate cortex (Liu et al., 2011). Using the whole left and right cerebellum as seeds treats the cerebellum as functionally homogenous throughout, an approach that can detect global anomalies but may miss and cannot define anomalies related to the known substructure of the cerebellum. In SZ and non-psychotic siblings of SZ patients, Repovs and colleagues examined the FC of spherical ROI's within four resting state networks, including default mode, frontoparietal, cingulo-opercular, and cerebellar networks (Repovs et al., 2011). They found that three of four ROI's in the cerebellar network had reduced FC with the frontoparietal network, and that all four cerebellar ROI's had reduced FC with the cingulo-opercular network in SZ (Repovs et al., 2011). Again, however, restricting FC analysis to the four ROI's in the so-called cerebellar network provides valuable information but excludes the majority of the cerebellum's functional topography.

Finally, Chen and colleagues examined cerebellar FC in SZ with seeds chosen as the two peak cerebellar coordinates of six large scale networks determined using independent component analysis (Chen et al., 2013). These authors observed decreased FC between cerebellum and frontal, parietal, and cingulate regions when seeding from cerebellar regions associated with cingulo-opercular, right frontoparietal, dorsal default mode, and motor networks; and increased FC between cerebellum and sensorimotor regions when seeding from cerebellar regions associated with cingulo-opercular, left frontoparietal, and dorsal default mode networks (Chen et al., 2013). In addition, this group found reduced FC between cerebellum and thalamus in all networks except the motor network (Chen et al., 2013).

In all of these studies, researchers examined connectivity patterns associated with seeds originating in the cerebellum. Importantly, other than the study by Chen et al. (2013), these studies used parcellation schemes that were not based on intrinsic network organization. To date, no studies have examined the topographic representations of cerebro-cerellar resting state network abnormalities within the cerebellum in SZ. Here, we systematically investigated cerebral cortical-cerebellar FC in SZ and healthy control (HC) subjects by comparing connectivity between cerebellum and 10 cerebral cortical networks from Yeo et al.'s 17-network parcellation solution (Yeo et al., 2011). These are intrinsic large-scale networks, and thus can inform about differences between SZ and HC in many aspects of cerebellar functional network topography. Given the functional and topographical heterogeneity of the cerebellum and its connections with the cerebral cortex, we hypothesized that cerebral cortical-cerebellar connectivity would be abnormal in highly selective ways, not just in a generalized pattern, with networks differentially showing increased, decreased, or no differences in connectivity relative to healthy control participants depending on the functional role(s) of each network. Given the predominant cognitive, behavioral, affective, and perceptual clinical abnormalities in SZ relative to the more subtle motor abnormalities in SZ, we expected that higher-level association networks would be preferentially affected. Furthermore, we anticipated that network abnormalities in the cerebellum would mirror those that have been found in the cerebral cortex of SZ patients (e.g., as described in Baker et al., 2014). Thus, we predicted greatest disruption of cerebral cortical-cerebellar FC in the frontoparietal control networks.

Before describing the results, some comments on terminology and interpretation are needed: The cerebrum consists of both cerebral cortex and subcortical regions. Our FC seeds consist of only cerebral cortical and no subcortical regions. However, in the remainder of this paper, we will use the term “cerebro-cerebellar FC” rather than “cerebral cortical-cerebellar FC” both for simplicity and because subcortical regions are inherent components of these intrinsic networks even if our FC seeds did not specifically include them.

## Materials and methods

### Participants

We studied 44 patients with schizophrenia, schizoaffective disorder, or schizophreniform disorder—whom we will collectively refer as the schizophrenia (SZ) group—and 28 healthy controls (HC). Participants were men and women, ages 18–65 years. We recruited patients from inpatient and outpatient services at McLean Hospital, and HC through community advertisements. This study was approved by the McLean Hospital Institutional Review Board and all participants provided written informed consent. We administered the Structured Clinical Interview for DSM-IV-TR (SCID) (First et al., 1995) to confirm diagnoses in the patient group, and to rule out current axis I psychiatric disorders in HC. We excluded individuals with substance abuse or dependence in the previous 3 months, electroconvulsive treatment in the past year, or major medical or neurological illnesses that could contribute to patients' psychiatric presentation or brain function. We matched the two groups for mean age and sex.

Data from 38 of the 44 patients and all 28 HC in this study were included in a study investigating auditory hallucinations previously published by our group (Shinn et al., 2013a). The auditory hallucinations dataset consisted of 41 patients; we excluded three of these 41 patients because their resting fMRI images did not capture the cerebellum in its entirety. Additionally, there are six patients included in the current analysis who were not included in the original auditory hallucinations study because their data were acquired after data analysis for the auditory hallucinations report had begun.

Participants underwent a comprehensive evaluation, including the Scale for the Assessment of Negative Symptoms (SANS) (Andreasen, 1983) and Positive Symptoms (SAPS) (Andreasen, 1984), the Young Mania Rating Scale (YMRS) (Young et al., 1978), the Montgomery-Asberg Depression Rating Scale (MADRS) (Montgomery and Asberg, 1979), the auditory hallucinations subscale of the Psychotic Symptom Rating Scale (PSYRATS-AH) (Haddock et al., 1999), and the Fagerstrom Smoking Questionnaire (Heatherton et al., 1991). When administering the scales, we asked patients to focus on the most severe symptoms within the previous month. SAPS, SANS, and Fagerstrom data were unavailable for a subset of 12 patients. The PSYRATS-AH data were not available for eight patients. We collected medication information, and calculated chlorpromazine (CPZ) equivalent doses for antipsychotic medications. All but two patients were taking antipsychotics at the time of study participation; none were antipsychotic-naïve.

### Image acquisition

Using a Siemens Trio 3-Tesla MRI scanner, we acquired a T1-weighted whole-brain anatomical image (MPRAGE, 256 × 256 voxels, 1 × 1.3 mm^2^ in-plane resolution, 1.3 mm slice thickness), followed by a T2-weighted functional scan (interleaved EPI sequence, 42 oblique slices, flip angle 82°, TE/TR = 24/2500 ms, 3.5 mm isotropic voxels, matrix 128 × 128, 224 mm^2^ FOV). We acquired 240 volumes over 10 min. Participants were scanned at rest, and instructed to stay awake, keep their eyes open, and think of nothing in particular.

For quality control, we assessed between-group differences in head motion (mean absolute displacement of each brain volume compared to the previous volume) (Jenkinson et al., 2002; van Dijk et al., 2012).

The scanner at the McLean Hospital Brain Imaging Center underwent an upgrade to total imaging matrix (TIM) technology in September 2009, during the data collection period. Twenty-eight of the 72 subjects (17/44 SZ and 11/28 HC) were scanned post-upgrade. However, scan parameters were identical for all subjects, and as we showed in our previous paper (Shinn et al., 2013a), there were no statistically significant differences in image quality pre- and post-upgrade.

### Image analysis

We used the FMRIB Software Library (FSL v4.1.6) (Smith et al., 2004) for image analyses. We discarded the first four volumes of the resting BOLD image to account for magnet stabilization. Images were slice-time and motion corrected (Jenkinson et al., 2002), smoothed with a 6 mm Gaussian kernel, and affine registered to Montreal Neurological Institute (MNI) space. Images were low-pass filtered at 0.08 Hz (sigma = 22.2 volumes) to reduce frequency non-neuronal noise most likely due to cardiac and respiratory artifacts, and high-pass filtered at 0.009 Hz (sigma = 2.5 volumes) to remove low-frequency scanner drift.

**Figure 1:**
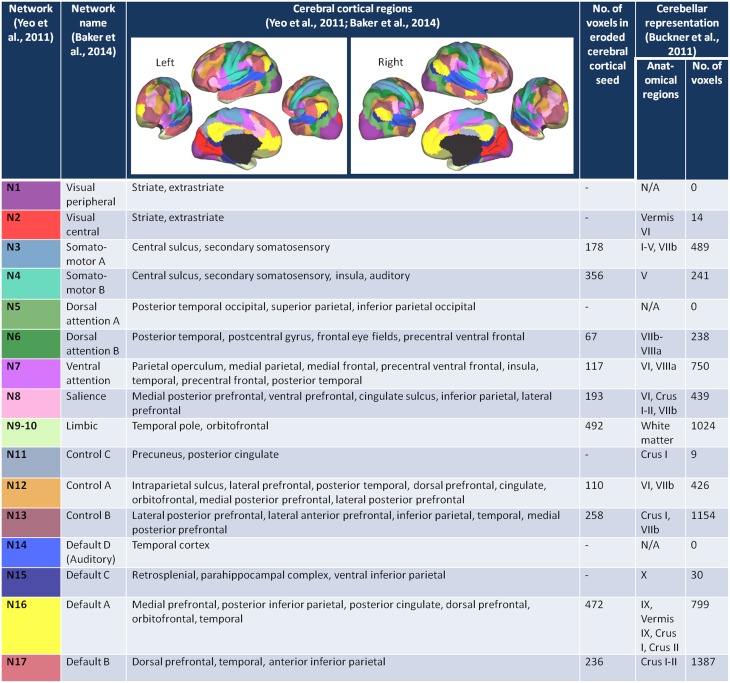
**The cerebral cortex network seeds and their cerebellar representations**. The figures of the left and right 17-network parcellation of the human cerebral cortex are adapted with permission from Yeo et al. (2011), p. 1139, Figure 13. The network names and the cerebral cortical regions that compose the 17 networks are from the supplementary video in Baker et al. (2014). The number of voxels in each of the eroded cerebral cortical seeds was determined after the (Yeo et al., 2011) 17-network cortical parcellation was resampled from 1 to 2 mm space, segmented into individual network maps, and eroded (see Materials and Methods). The number of voxels within each of the cerebellar network maps (Buckner et al., 2011) was determined after the 17-network loose estimate of the cerebellum (publicly available at http://www.freesurfer.net/fswiki/CerebellumParcellation_Buckner2011) was resampled from 1 to 2 mm MNI space and segmented into individual network maps.

We started with the 17 network cortical parcellation map (Figure 1) derived from the fMRI data of 1000 healthy controls (Yeo et al., 2011), publicly available at http://surfer.nmr.mgh.harvard.edu/fswiki/CorticalParcellation_Yeo2011, as the basis for our FC seeds. We decided on the 17-network rather than 7-network parcellation map because the network clusters from the 7-network solution are large and we sought to minimize the risk of averaging time courses that may be sufficiently different from one another. We reoriented the 17-network cortical parcellation map to the Montreal Neurological Institute (MNI) 152 subject image in FSL and resampled the images into 2 mm voxel space. We segmented the 17-network image into 17 separate images (N1-N17), each containing a map of a single network.

The networks are named according to the naming scheme in Baker et al. (2014). Figure 1 lists the networks, the cortical regions that make up each of the networks, and the corresponding network representations within the cerebellum (Buckner et al., 2011). Six of the 16 networks—visual peripheral (N1), visual central (N2), dorsal attention A (N5), control C (N11), auditory (N14), and default mode C (N15) networks—had minimal (≤ 30 voxels) representation in the 17 network cerebellum maps published in Buckner et al. (2011), and we therefore excluded these networks from our analysis. To maintain consistency with Baker et al. (2014), we combined networks 9 (temporal pole) and 10 (orbitofrontal cortex) into a single limbic network. Thus, we analyzed cerebro-cerebellar connectivity for a total of 10 networks.

According to Yeo and colleagues, there is less confidence in the regions close to the boundaries between networks (Yeo et al., 2011). To capture the time course of regions with higher confidence of being in the assigned network, and thus avoid potential boundary contamination, we eroded each of the network maps by one voxel layer using a 3D kernel (see Figure 1 fourth column for the number of voxels in each eroded network seed).

For each of the 10 networks, the mean BOLD time course across all voxels within that network was extracted and entered into a general linear model (GLM) using FEAT (www.fmrib.ox.ac.uk/fsl/feat5), with signal from white matter, CSF, and motion correction parameters regressed out. Data from first-level analyses were entered into a mixed-effects group analysis using the Bayesian estimation techniques in FLAME (Woolrich et al., 2004). We generated group maps for SZ and HC, and performed between-group contrasts for SZ > HC and HC > SZ. Given our goal to investigate between-group connectivity differences in the cerebellum, we restricted our analysis to the cerebellum, using the Cerebellar Atlas in FSL as a mask. For the primary analyses, we entered age, sex, and chlorpromazine equivalents as covariates, and also controlled for the TIM scanner upgrade. To explore how antipsychotic medications might affect FC measures, we repeated the group analyses for all 10 networks, still controlling for age, sex, and scanner upgrade, but not controlling for chlorpromazine equivalents. For all group analyses, we used a *p* < 0.01 voxel threshold, corrected for multiple comparisons using a *p* < 0.05 cluster threshold.

To quantitatively assess the degree of overlap between the group cerebellar maps (SZ, HC) and the canonical cerebellar maps (Buckner et al., 2011) for each of the 10 networks, we calculated accuracy:

(1) $$\text{Accuracy}=\frac{\left(\text{True positive voxels}+\text{true negative voxels}\right)}{\left(\text{Positive voxels}+\text{negative voxels}\right)}$$

We identified *positive* voxels to be those within the canonical (Buckner et al., 2011) cerebellar map. *True positive* voxels were those that intersected between the group map and the canonical map. *Negative* voxels were those voxels within the cerebellum but outside the canonical cerebellar map. *True negative* voxels were calculated as the negative voxels minus false positive voxels, or those voxels in the group cerebellar map falling outside the canonical cerebellar map.

We also calculated sensitivity (true positive voxels/positive voxels) and specificity (true negative voxels/negative voxels) (see Supplementary Tables 1A–C).

### Correlation with symptom scales

For the five networks in which we found statistically significant between-group differences showing SZ hypoconnectivity and the two networks showing SZ hyperconnectivity, we extracted the BOLD time course for the masked clusters (with all clusters within a particular contrast combined in cases where there was more than one cluster in the findings). We calculated the Pearson's correlation coefficient between the averaged time course for each cluster and that of the cortical network seed. These Pearson's values were then correlated with the SAPS, SANS, YMRS, MADRS, and PSYRATS-AH in a matrix of five symptom scales and seven network contrasts. We considered this an exploratory analysis, as we did not collect symptom scales for the specific purpose of testing questions about cerebro-cerebellar FC. We used a significance threshold of *p*<0.001 (*p*<0.05 Bonferroni-corrected for 35 tests).

There were no statistically significant between-group differences in head motion, as measured by absolute mean displacement (in millimeters: SZ 0.36 ± 0.29, HC 0.32 ± 0.22; p = 0.50).

## Results

### Participants

The SZ and HC groups were comparable with respect to age, sex, and parental education level. Table 1 provides demographic and clinical details, as well as information about medications that patients were taking at the time of study participation.

**Table 1:** **Participant characteristics**.

	**SZ**	**HC**	**Statistic**	**Significance**
Sample size (*N* = 72)	44	28		
DSM-IV-TR diagnosis				
Schizophrenia, no. (%)	19 (43)			
Schizoaffective, no. (%)	23 (52)			
Schizophreniform, No. (%)	2 (5)			
Age, mean ± *SD*, y	38 ± 10 (20-57)	39 ± 9 (24-54)		*p* = 0.670
Female sex, no. (%)	18 (41)	11 (39)	χ^2^ = 0.019	*p* = 0.891
Parental education, No. (%)^a^	29 (66)	15 (55)	χ^2^ = 1.096	*p* = 0.295
Illness duration, mean ± *SD* (range), y	15 ± 11 (0-36)			
Inpatient hospitalized, No. (%)	27 (61)			
SAPS^b^	35.8 ± 19.1			
SANS^b^	23.5 ± 15.9			
YMRS	14.7 ± 8.0			
MADRS	14.4 ± 9.3			
PSYRATS-AH^c^	12.7 ± 14.3			
Fagerstrom^b^	1.2 ± 2.9			
Chlorpromazine Equivalent	580 ± 618 mg			
Antipsychotic-Free, No. (%)^d^	2 (5)			
Typical Antipsychotic, No. (%)	7 (16)			
Haloperidol	2 (5)			
Fluphenazine	1 (2)			
Perphenazine	3 (7)			
Loxapine	1 (2)			
Atypical Antipsychotic, No. (%)	39 (89)			
Clozapine	13 (30)			
Olanzapine	8 (18)			
Risperidone	8 (18)			
Paliperidone	1 (2)			
Quetiapine	7 (16)			
Aripiprazole	8 (18)			
Ziprasidone	1 (2)			
Antiepileptic, no. (%)	12 (27)			
Lithium, no. (%)	11 (25)			
Benzodiazepine, no. (%)	13 (30)			
Antidepressant, no. (%)	19 (43)			

a *At least one parent with a college degree*.

b *Data were missing for 12 patients*.

c *Data were missing for eight patients*.

d *No patients were antipsychotic-naïve*.

### Within-group maps

**Figure 2:**
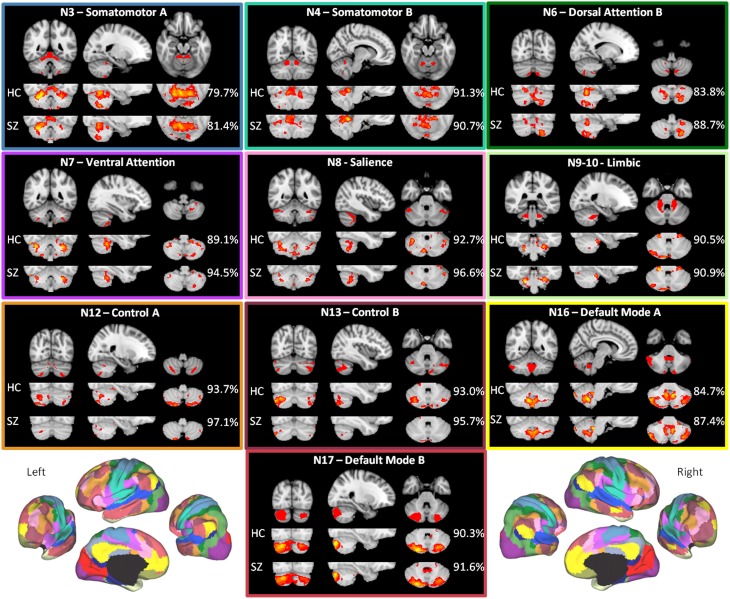
**The within-group maps capture the canonical cerebellar maps with high accuracy**. The canonical cerebellar maps (depicted in the uppermost rows) are from Buckner et al. (2011), in which data from 1000 healthy individuals were used to map every voxel within the cerebellum to its most strongly associated cerebral cortical network (Yeo et al., 2011) using a winner-take-all approach. The percentage to the right of each healthy control (HC) or schizophrenia (SZ) group map is the estimation of the map's spatial accuracy (= true positive voxels + true negative voxels)/(positive voxels + negative voxels), using the canonical (Buckner et al., 2011) map as the reference. For the purposes of display, the HC and SZ group maps were thresholded at a significance level of p<0.05, uncorrected. The boxes around each of the networks are highlighted to match the color coding of the networks in Figure 1. The figures of the left and right 17-network parcellation of the human cerebral cortex in the lower left and lower right, respectively, are adapted with permission from Yeo et al. (2011), p. 1139, Figure 13.

As shown in Figure 2, the HC group maps captured the canonical (Buckner et al., 2011) cerebellar maps with 90.1% mean accuracy (range 80.0–96.0%), a high degree of correspondence. For the HC group, accuracy was highest for somatomotor B, salience, limbic, control A, control B, and default mode B networks, all of which had greater than 90% accuracy. For these networks, there is a higher degree of confidence in the ability of the methods to detect regions in the cerebellum that are functionally coherent with their cerebral cortical seeds. The high accuracy measures appear to be driven primarily by high specificity (mean 89.9%, range 80.1–95.4%), which were greatest in control B, control A, salience, and limbic networks (Supplementary Table 1A). Sensitivity was comparatively lower (mean 33.7%, range 6.3–83.3%). The networks with the highest sensitivity in the HC group were default mode B and A, followed by somatomotor networks B and A (Supplementary Table 1A).

The mean accuracy for the SZ group maps was similarly high at 91.5% (range 81.4%–97.1%). As in the HC group, these values appear to be driven more by specificity (mean 92.7%, range 81.9–98.7%) than sensitivity (mean 24.9%, range 0.9–79.2%) (Supplementary Table 1B). The high correspondence between the SZ and canonical cerebellar maps suggests that the overall network organization within the cerebellum is relatively preserved in SZ.

### Between-group contrasts

#### Healthy control > schizophrenia

**Figure 3:**
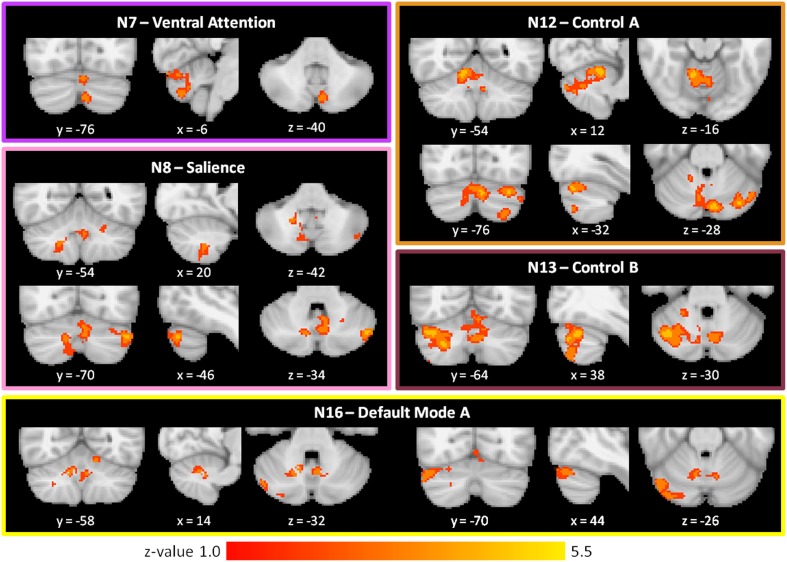
**Decreased cerebro-cerebellar functional connectivity in higher-level association networks in schizophrenia**. Regions in the posterior lobe of the cerebellum, especially Crus I and Crus II, are preferentially affected. These images were generated using a *p* < 0.01 voxel threshold, corrected for multiple comparisons using a *p* < 0.05 cluster threshold. The boxes associated with each network are highlighted to match the color coding of the networks in Figure 1.

As shown in Figure 3 and Table 2, we found SZ to have reduced cerebro-cerebellar functional connectivity (FC) in ventral attention (N7), salience (N8), control A (N12), control B (N13), and default mode A (N16) networks compared to HC. The cerebellar areas of greatest difference between SZ and HC were in left Crus II for the ventral attention network, left Crus I for the salience network, right lobule V (with extensions into left Crus I and bilateral Crus II) for the control A network, right Crus I for the control B network, and right Crus I for the default mode A network.

**Table 2:**
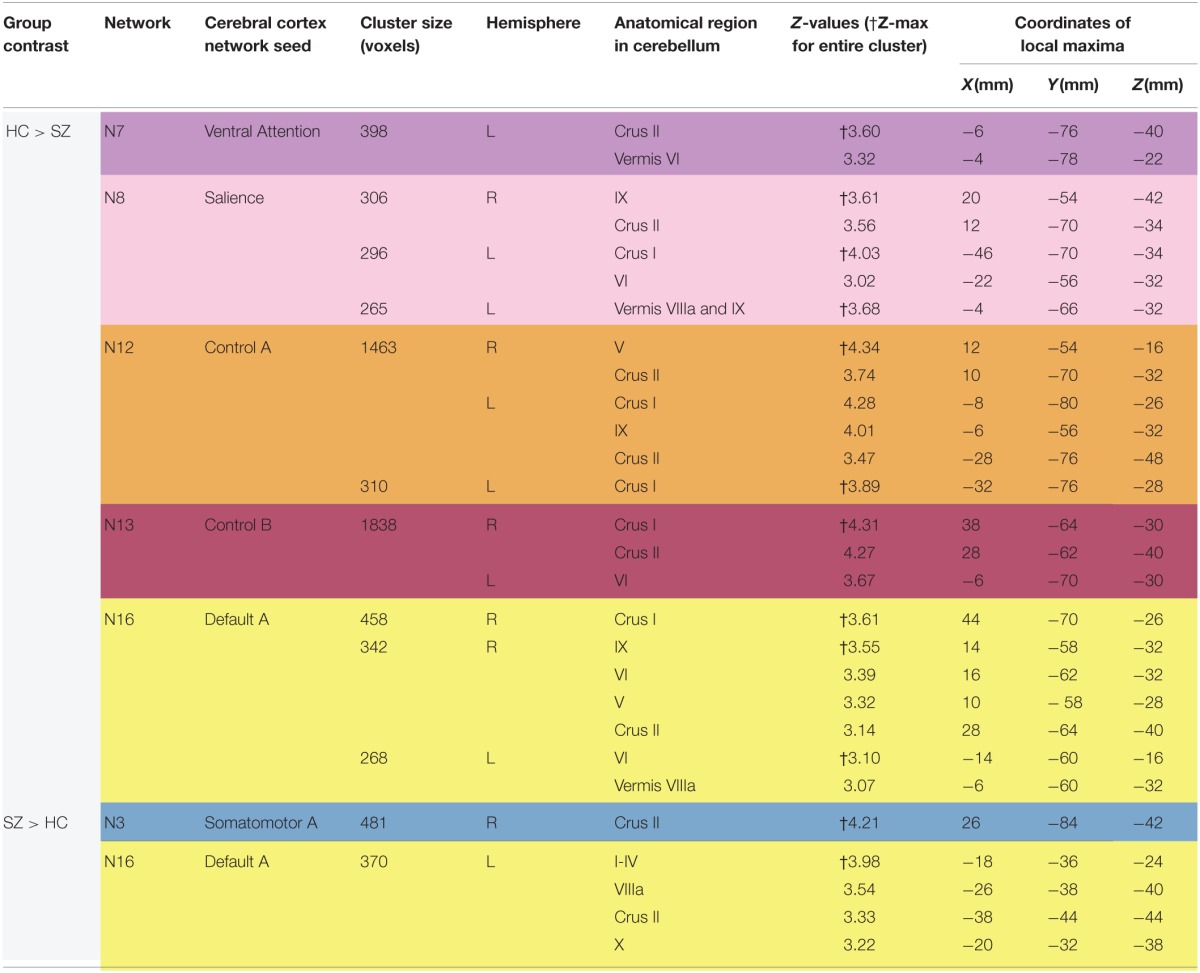
**Differences between schizophrenia and healthy controls in cerebro-cerebellar network connectivity**.

*Voxel threshold *p* < 0.01, cluster corrected at *p* < 0.05*.

*The results are color coded to match the network colors as shown in Figure 1*.

We found comparable results involving the same five networks (N7, N8, N12, N13, and N16) when not controlling for antipsychotic medications (Supplementary Table 2). However, we additionally detected reduced cerebro-cerebellar FC in the dorsal attention B network (N6); this finding localized to left Crus I and is relatively weak (cluster size 274 voxels and Z-max of 3.22) compared to the SZ hypoconnectivity findings in the five networks already described (Supplementary Table 2).

#### Schizophrenia > healthy control

**Figure 4:**
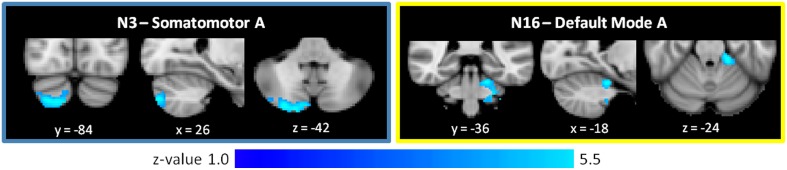
**Increased cerebro-cerebellar functional connectivity in somatomotor and default mode networks in schizophrenia**. The region that is hyperconnected with the somatomotor A network in SZ is in the posterior cerebellum and strongly resembles the cerebellar map of default mode network B. The region that is hyperconnected with the default mode A network in SZ is in the anterior cerebellum and corresponds topographically to somatomotor areas of the cerebellum. Together, these findings suggest that the somatomotor and default mode networks might be abnormally linked with one another in SZ. These images were generated using a p < 0.01 voxel threshold, corrected for multiple comparisons using a p < 0.05 cluster threshold. The boxes associated with each network are highlighted to match the color coding of the networks in Figure 1.

As shown in Figure 4 and Table 2, the SZ group, relative to HC, showed greater cerebro-cerebellar FC in a posterior component of the somatomotor A (N3) network and in an anterior component of the default mode A (N16) network. As just described, the default mode A network was also found to have decreased connectivity in SZ relative to HC. Importantly, the cluster associated with default mode hyperconnectivity does not overlap spatially with clusters in N16 found to be hypoconnected in SZ.

Notably, the cluster associated with default mode hyperconnectivity in SZ is located in somatomotor regions of the cerebellum (I–IV), corresponding roughly to where the lower limbs of the body (e.g., foot) tend to be topographically represented in the cerebellum. Furthermore, the cluster associated with somatomotor hyperconnectivity in SZ is located in Crus II, in a pattern resembling the default mode group cerebellar map (N17). This mirroring of FC patterns within the somatomotor and default mode networks suggests that somatomotor and default mode networks may be inappropriately coupled to one another in SZ.

SZ did not differ from HC in the cerebro-cerebellar FC of somatomotor B (N4), dorsal attention B (N6), limbic (N9-10), and default mode B (N17) networks in our subjects.

When we explored these results not controlling for antipsychotic medications, the somatomotor (N3) and default mode (N16) hyperconnectivity findings did not meet our specified significance threshold of voxel p < 0.01, cluster corrected at p < 0.05 (Supplementary Table 2). However, we found that trends for both the original medication-corrected N3 and N16 findings were present when we performed an additional post-hoc exploratory analysis using a statistical threshold of p < 0.05, uncorrected. Specifically, in the default mode A (N16) network, SZ patients showed hyperconnectivity in an anterior portion of the cerebellum, corresponding to somatomotor representations (Supplementary Figure 1C). And in the somatomotor (N3) network, SZ patients showed hyperconnectivity in right Crus II, in a pattern resembling the topography of the default mode B (N17) network (Supplementary Figure 1A). Intriguingly, in this multiple comparisons uncorrected, exploratory analysis of N3, we also observed clusters strongly reminiscent of the topographical pattern of default mode A (N16) (Supplementary Figure 1B), which is possibly suggestive of increased connectivity between somatomotor and default mode networks in general (not just default mode network A or B).

### Correlation with symptoms

There were no statistically significant correlations between FC for any of the seven network that showed between-group findings and the five symptom scales obtained (SAPS, SANS, YMRS, MADRS, PSYRATS-AH) (Supplementary Table 3). We did not test for correlations to specific symptoms within the scales, as this would entail a very large number of comparisons and, therefore, obtaining significant results after correction would require a much larger sample. Nor did we test for correlations to specific cognitive deficits or motor abnormalities, which would require a much more detailed evaluation of each subject.

## Discussion

As noted, the cerebellum participates in non-motor as well as motor functions, and it has been proposed that the diverse and wide-ranging disturbances in SZ may be manifestations, in part, of uncoordinated mental activity resulting from cerebellar dysfunction or disconnection (Andreasen et al., 1998; Schmahmann, 1998). To investigate cerebellar network abnormalities in SZ, we used 10 well-defined cerebral cortical networks (Yeo et al., 2011) as the seeds for resting state functional connectivity (FC) analysis within the cerebellum. We found SZ patients to have decreased cerebro-cerebellar FC in higher level association networks (ventral attention, salience, control, and default mode networks) relative to healthy control participants. We also observed SZ patients to have increased cerebro-cerebellar connectivity in somatomotor and default mode networks. The cerebellar regions showing default mode hypoconnectivity in SZ did not overlap with those found to be hyperconnected within the same default mode network. Lastly, we found evidence to suggest that somatomotor and default mode networks may be inappropriately linked in SZ patients.

Our findings support several of our predictions. The observation that SZ patients have reduced cerebro-cerebellar FC in association networks, especially frontoparietal control networks, is consistent with both the cardinal (e.g., hallucinations, delusions, irrational thoughts, disorganized behavior, and negative symptoms) and broader (e.g., altered mood, cognitive and social dysfunction, and anomalous self-experiences) clinical features of the illness. Functional hypoconnectivity in association networks is also consistent with our prediction that abnormalities in the cerebellum would generally mirror those found in the cerebral cortex (Baker et al., 2014). The presence of such mirroring between the cerebral cortex and cerebellum is not surprising, given the reciprocally interconnected architecture of cerebro-cerebellar circuitry, and highlights the importance of considering representations in the cerebellum as functional entities within large-scale networks.

Our observation of somatomotor cerebro-cerebellar hyperconnectivity, on the other hand, was initially unexpected given that motor impairments are not usually considered a central feature of SZ. An abnormality of the somatomotor network was also not predicted by our hypothesis that cerebellar anomalies would mirror cortical abnormalities, as our recent study of cortical network architecture in psychosis patients identified no specific deficits involving somatomotor networks (Baker et al., 2014). When considered further, however, the finding of somatomotor network abnormalities in the cerebellum is not inconsistent with the clinical picture of SZ. While motor symptoms are not considered cardinal in the definition of SZ, neurological soft signs are highly prevalent in SZ (with at least one neurological soft sign detectable in 98% of SZ patients Lane et al., 1996) In fact, neurological soft signs are detectable prior to the onset of frank psychosis and in well relatives of patients with SZ, and may be associated with poor premorbid adjustment and chronicity (Gupta et al., 1995; Keshavan et al., 2008; Prasad et al., 2009). Thus, neurological soft signs, while not specific to SZ, are as much a feature of SZ as any other signs or symptoms, and altered FC in somatomotor networks might have been expected. That being said, it is unclear how to interpret the direction of the somatomotor network dysconnectivity. That we observed increased cerebellar connectivity in a somatomotor network is compatible with the concept of neuromotor symptoms in SZ. According to Schmahmann, cerebellar lesions can partly be understood as a problem of timing and adjustment; the cerebellum “regulates the rate, force, rhythm, and accuracy of movements” and other mental processes, and there can be both overshoot or undershoot (Schmahmann, 1998). Though speculative, increased somatomotor FC may reflect a dysfunctional network that has deviated from its cerebellum-mediated homeostatic baseline.

Neurological soft signs represent a developmental phenomenon, with higher prevalence in childhood and improvement during adolescence (Zabala et al., 2006). Persistence of neurological soft signs beyond adolescence is believed to represent abnormal development (Whitty et al., 2009). Our finding of increased FC in a somatomotor network in SZ, in this context, may reflect abnormal brain development, especially during the critical period of transition to adulthood, a period in which overt psychosis is most likely to appear in susceptible individuals. Consistent with this possibility, Woodward and colleagues interpreted their finding of increased motor/somatosensory-thalamocortical FC as possibly relating to abnormal brain maturation interfering with the refinement of somatomotor-thalamic connectivity during the transition from adolescence to adulthood (Woodward et al., 2012). We did not measure abnormal movements in our patients, and the relationship between neurological soft signs and other sensorimotor impairments could be directly assessed in future studies.

Importantly, while our finding of increased cerebro-cerebellar FC in a somatomotor network does not mirror the disruptions described in the cortical networks of SZ patients, the combination of hypoconnectivity in association networks and hyperconnectivity in a somatomotor network is generally consistent with a previous study of altered cerebellar FC in SZ (Chen et al., 2013), and also complements two independent findings of reduced prefrontal-thalamic connectivity and increased motor/somatosensory-thalamic connectivity in SZ (Woodward et al., 2012; Anticevic et al., 2014). As described in the introduction, the cerebro-cerebellar circuit is polysynaptic, with feed-forward projections from cerebellum including the pontine nuclei and feedback projections involving the thalamus. The recurrence of the same pattern of disturbance in different segments of the cerebro-cerebellar circuit suggests that the abnormality is widely distributed. On the other hand, increased FC in somatomotor networks, to our knowledge, has not been found in the literature on cortical FC abnormalities in SZ, and this may suggest that the cerebral cortex is somehow less vulnerable to or can compensate for this dysfunction.

In interpreting our findings, it should be noted that the cerebellum has regional and functional specificity. The cerebellar cortex can be subdivided along the anterior-posterior plane into the anterior lobe, the posterior lobe, and the flocculonodular lobe (phylogenetically the oldest part of the cerebellum, thus also called the archicerebellum). It can also be subdivided medial-laterally into the vermis (Latin for “worm,” located along the mid-sagital plane of the cerebellum) and paravermis (which together make up the paleocerebellum), and the lateral hemispheres (neocerebellum). Clinical studies have suggested that the anterior lobe of cerebellum is primarily engaged in motor control, while the vermis is involved in affective processing and the posterior lobe is involved in cognitive functions (Stoodley and Schmahmann, 2009). A meta-analysis of functional neuroimaging studies similarly indicates that sensorimotor tasks activate anterior lobes (lobules V, VI, VIII), while posterior lobes (lobules VI–VII and Crus I) are involved in higher-level tasks such as language, verbal working memory, executive functions, and emotional processing (Stoodley and Schmahmann, 2009). That the majority of our findings of hypoconnectivity tend to involve more posterior aspects of the cerebellum, especially Crus I and Crus II, is in accordance with the well-documented disruptions in higher-level mental activity in SZ, including impairments in working memory, language, and emotional processing (Gold and Harvey, 1993; Reichenberg and Harvey, 2007).

We observed that the cluster that is hyperconnected with the default mode A network in SZ is in the anterior cerebellum and corresponds topographically to somatomotor areas of the cerebellum. Similarly, the cluster found to be hyperconnected with the somatomotor A network in SZ is in Crus II of the posterior cerebellum and strongly resembles the cerebellar map of a default mode network. Taken together, these findings suggest that the somatomotor and default mode networks might be abnormally linked, with decreased modularity, in SZ. This finding is intriguing. A network has high modularity when its component nodes are densely intra-connected to one another but only sparsely connected to nodes in other modules (Newman and Girvan, 2004; Newman, 2006b; Meunier et al., 2010). That is, a network with higher modularity is one that is more segregated. The healthy human brain is hierarchically modular, and such an organization facilitates segregated processing of specialized functions, while also enabling dynamic assembly of networks for more integrated activity (Meunier et al., 2010). Sensory-motor brain areas, in particular, are organized in serial, hierarchical pathways, with local connections predominating over distant ones (Meunier et al., 2009; Sepulcre et al., 2010; Buckner and Krienen, 2013). In the healthy brain, this modular organization extends to the cerebellum. Cerebro-cerebellar functional connectivity of motor vs. prefrontal cortical seed regions shows clear anatomic dissociation (Krienen and Buckner, 2009), and the cerebro-cerebellar circuits supporting sensory-motor functions are segregated from those that mediate cognitive functions (Salmi et al., 2009). Such modularity of motor and prefrontal functions in the cerebellum is consistent with the structural architecture of the cerebro-cerebellar system, in which closed circuit loops projecting to primary motor cortex are distinct from those projecting to prefrontal cortex (Kelly and Strick, 2003).

There is growing evidence that disruptions of modularity may play a role in the pathophysiology of SZ. Several studies report alterations of modularity in cerebral cortical regions (e.g., Alexander-Bloch et al., 2010; Yu et al., 2012; Baker et al., 2014; de Arruda et al., 2014). Recently, disrupted modularity in SZ was also reported in the cerebellum. Kim et al. (2014) performed graph theoretical analysis of diffusion tensor imaging data, and found SZ patients to have disrupted modular architecture despite overall intact global network properties (e.g., retained small; worldness). The disruption in architecture mainly involved Crus II—whereas in healthy control participants, Crus II belonged to modules containing other neighboring regions, the hemispheric and vermal Crus II regions formed their own distinct module in SZ (Kim et al., 2014).

Our observation of cerebro-cerebellar functional hyperconnectivity between the somatomotor and default mode networks in SZ may provide support for the “dysmodularity” model of SZ (Fodor, 1983; David, 1994), which proposes that SZ symptoms arise from excessive connectivity between otherwise modular regions, leading to a breakdown in “informational encapsulation,” domain specificity, and functional specialization. Put crudely, there is “cross-talk” where there should not be, and information “leaks” among the systems designed to isolate data processing. While speculative, the finding that somatomotor networks are less segregated and more connected with association networks in SZ relative to healthy controls may underlie some phenomenologic features of psychosis. In delusions of control, which some consider pathognomonic for SZ, the individual experiences a loss of agency and subjectively feels that he or she is being controlled by some outside force. Delusions of control can manifest as a loss of physical or bodily agency (e.g., an alien force controlling one's body, behavior, actions), or as a loss of mental or cognitive agency (e.g., thoughts being inserted, withdrawn, or broadcast aloud without one's control). Such “made” or “passivity” experiences, in which there is a blurring of the boundary between self and other, are common, occurring in 57–66% of SZ patients (Shinn et al., 2013b). The default mode network is implicated in self-referential (e.g., reminiscences of autobiographical memories) as well as in undirected, spontaneous mental activity (e.g., daydreaming and mind-wandering) (Andreasen et al., 1995; Gusnard et al., 2001; Buckner et al., 2008). It is plausible that dysmodularity between somatomotor and default mode networks could give rise to the experience of blurred sense of self agency as relates to somatosensory and motor control.

Embodied cognition, a class of theories from cognitive science that explore and explain how sensorimotor experience gained through bodily interactions with the environment relate to the acquisition and representation of conceptual knowledge (Wellsby and Pexman, 2014), may be relevant to understanding the altered sense of self experienced by patients with SZ. Action-perception or sensory-motor coupling may give an organism the concrete and continuous sense of inhabiting its body, and is necessary to distinguish activity that is self- vs. externally generated (Gapenne, 2014). According to a recent study of action-perception coupling in patients with focal cerebellar lesions, intact action-perception coupling depends on the integrity of the cerebellum (Christensen et al., 2014). Lesions that significantly correlated with impaired action-perception coupling were in ventral dentate nucleus, motor representations in lobules V and VI, and posterior cerebellum including Crus II (Christensen et al., 2014). Our SZ hyperconnectivity findings show a similar pattern, and point to the possibility of cerebellum-mediated action-perception abnormalities in SZ. Anomalous experiences involving bodily awareness and self-world boundaries have been described early in the SZ prodrome (Parnas, 1999; Parnas et al., 2005) and, in more severe forms, characterize the experience of florid first rank psychotic symptoms. Future studies should more directly investigate the relationship of symptoms involving self and bodily integrity and abnormalities in cerebro-cerebellar circuitry.

More broadly, our results, which show specific abnormal cerebro-cerebellar FC point to the cerebellum and its connections as potential targets for further elucidation of the underlying mechanisms producing symptoms of SZ. Daskalakis and colleagues have demonstrated that FC between the cerebellum and cerebral cortex in humans can be studied with transcranial magnetic stimulation (TMS) (Daskalakis et al., 2004). In particular, using a specific TMS paradigm (Daskalakis et al., 2004), this group showed that SZ patients have reduced cerebellar inhibition of TMS-induced motor evoked potentials compared to healthy subjects, suggesting that cortical inhibitory dysfunction in SZ might be mediated, in part, through cerebellar or cerebellar-thalamic-cortical connectivity abnormalities (Daskalakis et al., 2005). More recently, Halko and colleagues combined cerebellar TMS with rsfMRI to show that stimulating the human lateral cerebellar Crus I/II modulates the cerebral default mode network, while stimulation of vermal lobule VII changes the cerebral dorsal attention network (Halko et al., 2014). The safety, tolerability, and clinical promise of intermittent theta-burst stimulation of the cerebellar vermis using MRI-guided TMS in a small group of treatment refractory SZ patients has been demonstrated (Demirtas-Tatlidede et al., 2010). It would be intriguing to see if cerebellar TMS could normalize the hypoconnectivity of association networks and hyperconnectivity of somatomotor and default mode networks that we observed in our SZ sample. Such an effect would have implications for the role of the cerebellum and its connections as targets for treatment interventions (Hoppenbrouwers et al., 2008).

The findings of this study must be considered in the context of several limitations. First, the data were not specifically acquired for the purpose of investigating cerebro-cerebellar connectivity. We performed comprehensive clinical characterization (to include positive and negative symptoms, depression and mania scales, and also a specific scale to more fully characterize auditory hallucinations), but lacked specific measures of cognitive and motor (e.g., neurological soft signs) functioning. Thus, we were unable to test whether individual cerebro-cerebellar network abnormalities correlate with cognitive and motor symptoms in SZ. On the other hand, the “dysmetria of thought” and “cognitive dysmetria” models relate SZ symptoms in general, not just specific motor or cognitive abnormalities, to cerebellar dysfunction. Second, our sample size was modest, and our findings should be replicated in larger studies. Nonetheless, the sample was larger than those of most previous publications on cerebellar FC. Third, while the accuracy of the group maps in capturing the canonical Buckner maps was high, with good specificity, the sensitivity measures were relatively low. This could be due to different methods of preprocessing compared to Buckner et al. (2011). Moreover, Buckner et al. used a winner-take-all strategy in identifying the network membership of each cerebellar voxel, while this study did not. Fourth, we interpret our somatomotor and default mode hyperconnectivity results as suggestive of “dysmodularity” in SZ as conceptualized by David (1994). However, the term modularity in the neuroimaging field is more often connected with mathematical and computer algorithmic approaches to detect and quantify community structure (e.g., graph partitioning or hierarchical clustering) (Newman, 2006a,b), which we did not apply here. Future studies may wish to employ graph theoretical and other mathematical methods to more systematically characterize modularity and other properties of community structure (e.g., path length, clustering, small worldness, etc.) in cerebro-cerebellar functional networks.

Lastly, the majority of our SZ patients were medicated, and medication effects do appear to affect the functional connectivity results to some degree. In specific, when exploratory group analysis is performed without controlling for chlorpromazine equivalent doses of antipsychotic medications, we observe a new SZ hypoconnectivity finding in the dorsal attention B (N6) network. That this finding is not present in our primary analyses (including chlorpromazine equivalent doses in our model) suggests that this specific finding may be an effect of medications. By contrast, the SZ hyperconnectivity findings in the somatomotor (N3) and default mode A (N16) networks fail to meet statistical significance when we do not control for antipsychotic medications. The fact that these findings are stronger when medication-corrected and weaker when medication uncorrected suggests that the somatomotor and default mode hyperconnectivity findings may be true concomitants of illness that become partially normalized with antipsychotic treatment. Furthermore, the fact that we observe statistical trends for both the somatomotor and default mode hyperconnectivity findings even without correction for medication gives us confidence in the validity of the corresponding medication-corrected findings. In general, we have placed greater emphasis on and interpret findings from group analyses controlling for antipsychotic medications, given that we are mainly interested in illness rather than medication effects.

In conclusion, we provide further evidence in support of the “cognitive dysmetria” and “dysmetria of thought” models of SZ, which implicate aberrant cerebro-cerebellar circuitry involving non-motor as well as motor networks in the pathophysiology of SZ. The results we obtained show clear indications of specific alterations, both increases and decreases, in known connections between cerebellum and cerebral cortex. In particular, we show reduced cerebro-cerebellar FC of association networks. We also show increased cerebro-cerebellar FC of somatomotor and default mode networks, and evidence to suggest poor functional segregation, or “dysmodularity,” between these networks. The anomalies observed are consistent with the diversity of symptoms observed in SZ, including the sensorimotor abnormalities that are present in the disorder. Future studies may clarify the relation of the functional dysconnectivities to specific symptoms in SZ or relate them to subtypes of SZ. In addition, the results suggest the value of evaluating and perhaps even modifying cerebro-cerebellar circuitry through cerebellar TMS and other non-invasive brain stimulation modalities. Finally, our findings, taken together with the known modular, closed loop architecture of cerebro-cerebellar circuitry, suggest that the cerebellum ought to be considered for analysis in all future studies of network abnormalities in SZ.

## Author contributions

AKS collected the data, designed the study, analyzed and interpreted the data, and wrote the manuscript. JTB contributed to data analysis, interpretation, and drafting of the manuscript. KEL contributed to interpretation of the results and drafting of the manuscript. DO was involved in study design, data analysis, interpretation, and the drafting of the manuscript. BMC was involved in study design, interpretation of the results, and drafting of the manuscript. All authors approved the final manuscript version.

### Conflict of interest statement

The authors declare that the research was conducted in the absence of any commercial or financial relationships that could be construed as a potential conflict of interest.
