# Supplementary material for: Aberrant cerebellar connectivity in motor and association networks in schizophrenia
Source: Front Hum Neurosci. 2015 Mar 18;9:134. doi: 10.3389/fnhum.2015.00134 (PMC4364170; doi:10.3389/fnhum.2015.00134)
Supplement: Supplementary file 1 [file Table1.PDF]

**Abberant cerebellar connectivity in motor and association networks in schizophrenia (Shinn et al.)**
**Supplementary material: Calculation of group map accuracy, sensitivity, and specificity**

**Supplementary Table 1A. Healthy control group**

| | | | Positives (P) | True Positives (TP) | Sensitivity | Negatives (N) | False Positives (FP) | True negatives (TN) | Specificity | Accuracy |
|---|---|---|---|---|---|---|---|---|---|---|
| | Network | # Voxels in group map | # Voxels in Buckner Cerebellar Mask | #Voxels Overlap CombinedGrp-Buckner | #voxels overlap between group map & Buckner = TP/P | #non-Buckner cereb voxels | #voxels grp map minus #voxels overlap | = negatives minus false positives | = true negatives/negatives | = (TP +TN)/(P+N) |
| 1 | N3 | 7430 | 489 | 230 | 0.470 | 36174 | 7200 | 28974 | 0.801 | 0.797 |
| 2 | N4 | 3178 | 241 | 115 | 0.477 | 36422 | 3063 | 33359 | 0.916 | 0.913 |
| 3 | N6 | 5818 | 238 | 51 | 0.214 | 36425 | 5767 | 30658 | 0.842 | 0.838 |
| 4 | N7 | 3649 | 750 | 207 | 0.276 | 35913 | 3442 | 32471 | 0.904 | 0.891 |
| 5 | N8 | 2380 | 439 | 68 | 0.155 | 36224 | 2312 | 33912 | 0.936 | 0.927 |
| 6 | N9-N10 | 2591 | 1024 | 65 | 0.063 | 35639 | 2526 | 33113 | 0.929 | 0.905 |
| 7 | N12 | 2011 | 426 | 71 | 0.167 | 36237 | 1940 | 34297 | 0.946 | 0.937 |
| 8 | N13 | 1907 | 1154 | 256 | 0.222 | 35509 | 1651 | 33858 | 0.954 | 0.930 |
| 9 | N16 | 5599 | 799 | 392 | 0.491 | 35864 | 5207 | 30657 | 0.855 | 0.847 |
| 10 | N17 | 4466 | 1387 | 1156 | 0.833 | 35276 | 3310 | 31966 | 0.906 | 0.903 |
| Mean | | | | | 0.337 | | | | 0.899 | 0.889 |
| Min | | | | | 0.063 | | | | 0.801 | 0.797 |
| Max | | | | | 0.833 | | | | 0.954 | 0.937 |

**Abberant cerebellar connectivity in motor and association networks in schizophrenia (Shinn et al.)**
**Supplementary material: Calculation of group map accuracy, sensitivity, and specificity**

**Supplementary Table 1B. Schizophrenia group**

| | | | Positives (P) | True Positives (TP) | Sensitivity | Negatives (N) | False Positives (FP) | True negatives (TN) | Specificity | Accuracy |
|---|---|---|---|---|---|---|---|---|---|---|
| | Network | # Voxels in group map | # Voxels in Buckner Cerebellar Mask | #Voxels Overlap CombinedGrp-Buckner | #voxels overlap between group map & Buckner = TP/P | #non-Buckner cereb voxels | #voxels grp map minus #voxels overlap | = negatives minus false positives | = true negatives/negatives | = (TP +TN)/(P+N) |
| 1 | N3 | 6760 | 489 | 218 | 0.446 | 36174 | 6542 | 29632 | 0.819 | 0.814 |
| 2 | N4 | 3310 | 241 | 77 | 0.320 | 36422 | 3233 | 33189 | 0.911 | 0.907 |
| 3 | N6 | 3960 | 238 | 28 | 0.118 | 36425 | 3932 | 32493 | 0.892 | 0.887 |
| 4 | N7 | 1383 | 750 | 51 | 0.068 | 35913 | 1332 | 34581 | 0.963 | 0.945 |
| 5 | N8 | 907 | 439 | 48 | 0.109 | 36224 | 859 | 35365 | 0.976 | 0.966 |
| 6 | N9-N10 | 2527 | 1024 | 100 | 0.098 | 35639 | 2427 | 33212 | 0.932 | 0.909 |
| 7 | N12 | 648 | 426 | 4 | 0.009 | 36237 | 644 | 35593 | 0.982 | 0.971 |
| 8 | N13 | 495 | 1154 | 30 | 0.026 | 35509 | 465 | 35044 | 0.987 | 0.957 |
| 9 | N16 | 4630 | 799 | 400 | 0.501 | 35864 | 4230 | 31634 | 0.882 | 0.874 |
| 10 | N17 | 3885 | 1387 | 1098 | 0.792 | 35276 | 2787 | 32489 | 0.921 | 0.916 |
| Mean | | | | | 0.249 | | | | 0.927 | 0.915 |
| Min | | | | | 0.009 | | | | 0.819 | 0.814 |
| Max | | | | | 0.792 | | | | 0.987 | 0.971 |

**Abberant cerebellar connectivity in motor and association networks in schizophrenia (Shinn et al.)**
**Supplementary material: Calculation of group map accuracy, sensitivity, and specificity**

**Supplementary Table 1C. Combined healthy control and schizophrenia**

| | | | Positives (P) | True Positives (TP) | Sensitivity | Negatives (N) | False Positives (FP) | True negatives (TN) | Specificity | Accuracy |
|---|---|---|---|---|---|---|---|---|---|---|
| | Network | # Voxels in group map | # Voxels in Buckner Cerebellar Mask | #Voxels Overlap CombinedGrp-Buckner | #voxels overlap between group map & Buckner = TP/P | #non-Buckner cereb voxels | #voxels grp map minus #voxels overlap | = negatives minus false positives | = true negatives/negatives | = (TP +TN)/(P+N) |
| 1 | N3 | 7302 | 489 | 233 | 0.476 | 36174 | 7069 | 29105 | 0.805 | 0.800 |
| 2 | N4 | 3456 | 241 | 105 | 0.436 | 36422 | 3351 | 33071 | 0.908 | 0.905 |
| 3 | N6 | 5172 | 238 | 49 | 0.206 | 36425 | 5123 | 31302 | 0.859 | 0.855 |
| 4 | N7 | 2425 | 750 | 143 | 0.191 | 35913 | 2282 | 33631 | 0.936 | 0.921 |
| 5 | N8 | 1516 | 439 | 63 | 0.144 | 36224 | 1453 | 34771 | 0.960 | 0.950 |
| 6 | N9-N10 | 2690 | 1024 | 84 | 0.082 | 35639 | 2606 | 33033 | 0.927 | 0.903 |
| 7 | N12 | 1117 | 426 | 31 | 0.073 | 36237 | 1086 | 35151 | 0.970 | 0.960 |
| 8 | N13 | 1081 | 1154 | 115 | 0.100 | 35509 | 966 | 34543 | 0.973 | 0.945 |
| 9 | N16 | 5013 | 799 | 408 | 0.511 | 35864 | 4605 | 31259 | 0.872 | 0.864 |
| 10 | N17 | 4356 | 1387 | 1146 | 0.826 | 35276 | 3210 | 32066 | 0.909 | 0.906 |
| Mean | | | | | 0.304 | | | | 0.912 | 0.901 |
| Min | | | | | 0.073 | | | | 0.805 | 0.800 |
| Max | | | | | 0.826 | | | | 0.973 | 0.960 |
