# Supplementary material for: Aberrant cerebellar connectivity in motor and association networks in schizophrenia
Source: Front Hum Neurosci. 2015 Mar 18;9:134. doi: 10.3389/fnhum.2015.00134 (PMC4364170; doi:10.3389/fnhum.2015.00134)
Supplement: Supplementary file 2 [file Table2.PDF]

Supplementary Table 2. Differences between schizophrenia and healthy controls in cerebro-cerebellar network connectivity (*not* controlling for antipsychotic medications, as measured in chlorpromazine equivalent doses).

Group Contrast	Net-work	Cerebral Cortex Network Seed	Cluster Size (voxels)	Hemi-sphere	Anatomical Region in Cerebellum	Z-values (†Z-max for entire cluster)	Coordinates of Local Maxima		
							X (mm)	Y (mm)	Z (mm)
HC > SZ	N6	Dorsal Atten. B	274	L	Crus I	**†3.22**	-32	-86	-22
	N7	Ventral Attention	773	L	Crus II	**†4.00**	-2	-90	-22
					VI	3.85	-4	-78	-20
					Crus II	3.82	-4	-78	-40
					Vermis VI	3.42	-2	-84	-16
				R	VI	3.61	12	-82	-16
					Crus II	3.12	10	-70	-34
			317	L	VIIb	**†4.42**	-8	-40	-52
					IX	3.24	0	-46	-54
				R	VIIb	3.93	14	-38	-56
	N8	Salience	3241	R	Crus I	**†4.4**	42	-78	-30
				L	Vermis VI	4.33	-2	-76	-22
					VI	4.20	-10	-70	-26
					Crus I	4.20	-38	-66	-30
	N12	Control A	1223	L	Crus II	**†4.04**	-6	-80	-28
					V	3.80	-2	-58	-16
					VI	3.57	-16	-60	-22
				R	V	4.04	12	-52	-16
					Crus I	3.38	10	-70	-32
	N13	Control B	2836	R	Crus I	**†4.78**	38	-64	-28
					Crus II	4.31	44	-74	-46
					Crus I	3.67	44	-72	-38
				L	Crus I	3.42	-30	-74	-32
	N16	Default A	716	R	IX	**†3.95**	10	-58	-30
					Vermis VIIIa	3.18	2	-72	-46
				L	Vermis VIIIa	3.43	-6	-60	-32
			370	R	Crus I	**†3.63**	36	-82	-24
SZ > HC	*No findings.*								

Voxel threshold p < 0.01, cluster corrected at p < 0.05.
