# Supplementary material for: Aberrant cerebellar connectivity in motor and association networks in schizophrenia
Source: Front Hum Neurosci. 2015 Mar 18;9:134. doi: 10.3389/fnhum.2015.00134 (PMC4364170; doi:10.3389/fnhum.2015.00134)
Supplement: Supplementary file 3 [file Table3.PDF]

Supplementary Table 3. Correlations between network connectivity (Pearson's coefficient between cortical ROI and cerebellum findings) and clinical scales

	Network number	Network Name	SAPS (n=44)	SANS (n=44)	YMRS (n=44)	MADRS (n=44)	PSYRATS-AH (n=36)
**HC > SZ**	**N7**	Ventral Attention	0.244 *p*= 0.178	0.324 *p*=0.071	-0.66 *p*=0.669	0.118 *p*=0.444	0.120 *p*=0.485
	**N8**	Salience	0.031 *p*=0.868	0.098 *p*=0.593	0.222 *p*=0.147	0.109 *p*=0.480	-0.23 *p*=0.895
	**N12**	Control A	0.077 *p*=0.675	0.173 *p*=0.343	0.101 *p*=0.516	-0.006 *p*=0.969	-0.008 *p*=0.965
	**N13**	Control B	0.264 *p*=0.144	0.181 *p*=0.322	0.138 *p*=0.373	0.095 *p*=0.541	0.108 *p*=0.530
	**N16**	Default A	0.181 *p*=0.322	0.175 *p*=0.338	-0.138 *p*=0.370	0.027 *p*=0.862	0.000 *p*=0.998
**SZ > HC**	**N3**	Somatomotor	-0.180 *p*=0.323	0.329 *p*=0.066	-0.116 *p*=0.451	-0.031 *p*=0.844	-0.007 *p*=0.969
	**N16**	Default A	0.069 *p*=0.706	-0.178 *p*=0.330	0.068 *p*=0.660	0.059 *p*=0.702	-0.057 *p*=0.740

SAPS = Scale for the Assessment of Positive Symptoms (Andreasen, 1984); **SANS** = Scale for the Assessment of Negative Symptoms (Andreasen, 1983); **YMRS** = Young Mania Rating Scale (Young et al., 1978); **MADRS** = Montgomery-Asberg Depression Rating Scale (Montgomery and Asberg, 1979); **PSYRATS-AH** = Psychotic Symptom Rating Scale (Haddock et al., 1999), auditory hallucinations subscale.

Conclusion: We used a statistical significance threshold of $p < 0.001$ ($p < 0.05$ Bonferroni-corrected for 35 tests), and found no significant correlations between the imaging findings and the administered clinical scales.

References:

ANDREASEN, N. (1983) *Scale for the Assessment of Negative Symptoms (SANS),* Iowa City, University of Iowa.

ANDREASEN, N. C. (1984) *The Scale for the Assessment of Positive Symptoms (SAPS),* Iowa City, Iowa, University of Iowa.

HADDOCK, G., MCCARRON, J., TARRIER, N. & FARAGHER, E. B. (1999) Scales to measure dimensions of hallucinations and delusions: the psychotic symptom rating scales (PSYRATS). *Psychol Med,* 29**,** 879-89.

MONTGOMERY, S. & ASBERG, M. (1979) A new depression scale designed to be sensitive to change. *British Journal of Psychiatry,* 134**,** 382-389.

YOUNG, R. C., BIGGS, J. T., ZIEGLER, V. E. & MEYER, D. A. (1978) A rating scale for mania: reliability, validity and sensitivity. *Br J Psychiatry,* 133**,** 429-35.
