# Supplementary figures and images for: Aberrant cerebellar connectivity in motor and association networks in schizophrenia
Source: Front Hum Neurosci. 2015 Mar 18;9:134. doi: 10.3389/fnhum.2015.00134 (PMC4364170; doi:10.3389/fnhum.2015.00134)

# Supplementary Figures 1A-C

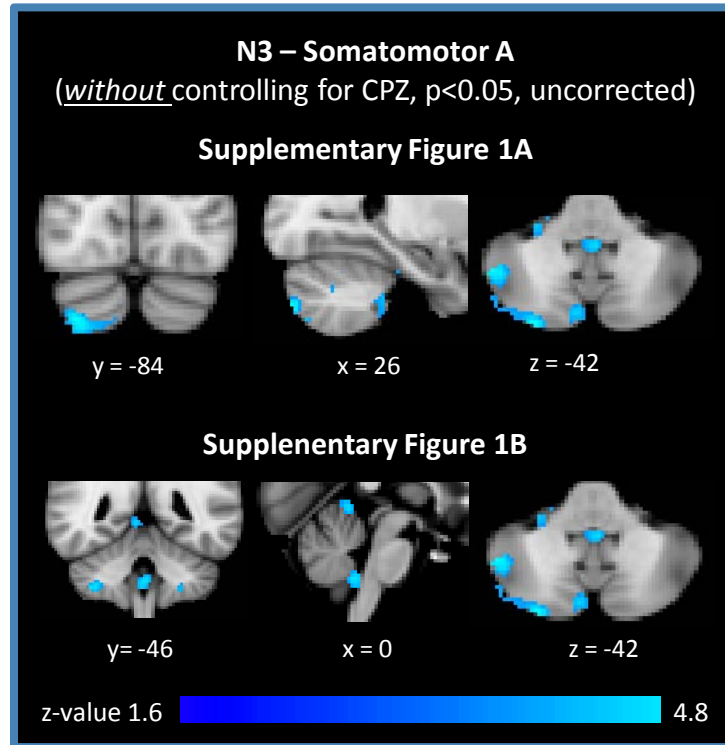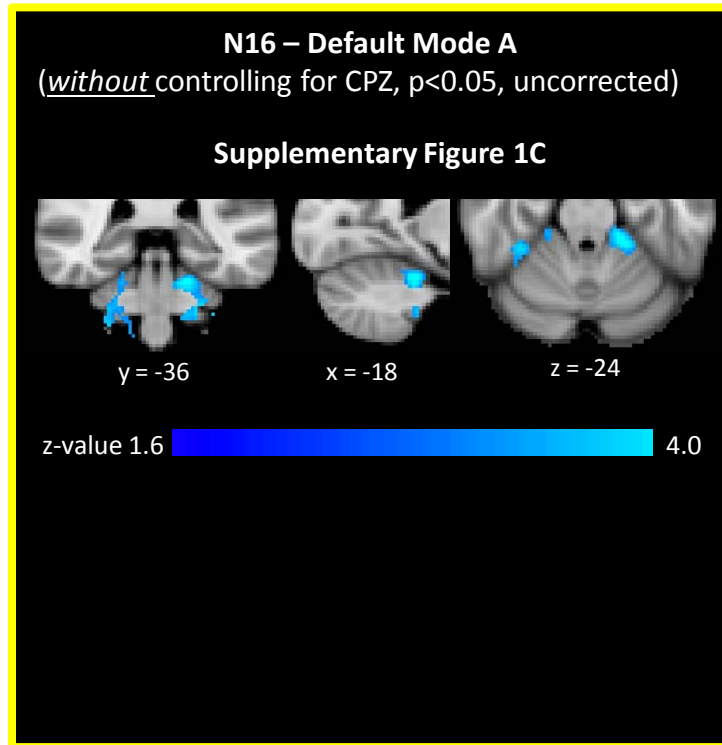

Supplement: Supplementary file 4 [file Image1.PDF]
